# Relation of *JAK2* V617F allele burden and coronary calcium score in patients with essential thrombocythemia

**DOI:** 10.2478/raon-2024-0036

**Published:** 2024-10-04

**Authors:** Ajda Drofenik, Ales Blinc, Mojca Bozic Mijovski, Tadej Pajic, Matjaz Vrtovec, Matjaz Sever

**Affiliations:** Department of Cardiology, Division of Internal Medicine, University Medical Centre Ljubljana, Ljubljana, Slovenia; Department of Vascular Diseases, Division of Internal Medicine, University Medical Centre Ljubljana, Ljubljana, Slovenia; Faculty of Medicine, University of Ljubljana, Ljubljana, Slovenia; Faculty of Pharmacy, University of Ljubljana, Ljubljana, Slovenia; Clinical Institute for Genomic Medicine, University Medical Centre Ljubljana, Ljubljana, Slovenia; Faculty of Medicine, University of Maribor, Maribor, Slovenia; Department of Dermatovenerology, University Medical Centre Ljubljana, Ljubljana, Slovenia; Department of Haematology, Division of Internal Medicine, University Medical Centre Ljubljana, Ljubljana, Slovenia

**Keywords:** essential thrombocythemia, *JAK2* V617F mutation, *JAK2* V617F allele burden, coronary calcium score

## Abstract

**Background:**

*JAK2* V617F (*JAK2*) mutation is associated with clonal hemopoiesis in myeloproliferative neoplasms as well as with faster progression of cardiovascular diseases. Little is known about the relationship between allele burden and the degree of atherosclerotic alteration of coronary vasculature. We previously reported that carotid artery stiffness progressed faster in patients with *JAK2* positive essential thromocythemia (ET) patients. After a four-year follow-up we investigated whether mutation burden of a *JAK2* allele correlates with a higher coronary calcium score.

**Patients and methods:**

Thirty-six patients with *JAK2* positive ET and 38 healthy matched control subjects were examined twice within four years. At each visit clinical baseline characteristics and laboratory testing were performed, *JAK2* mutation burden was determined, and coronary calcium was measured.

**Results:**

*JAK2* allele burden decreased in 19 patients, did not change in 5 patients, and increased in 4 patients. The coronary calcium Agatston score increased slightly in both groups. Overall, there was no correlation between *JAK2* allele burden and calcium burden of coronary arteries. However, in patients with the *JAK2* mutation burden increase, the coronary calcium score increased as well.

**Conclusions:**

The average *JAK2* allele burden decreased in our patients with high-risk ET during the four-year period. However, in the small subgroup whose *JAK2* mutation burden increased the Agatston coronary calcium score increased as well. This finding, which should be interpreted with caution and validated in a larger group, is in line with emerging evidence that *JAK2* mutation accelerates atherosclerosis and can be regarded as a non-classical risk factor for cardiovascular disease.

## Introduction

Essential thrombocythemia (ET) is one of the classic Philadelphia chromosome negative chronic myeloproliferative neoplasms (MPN), along with polycythemia vera and primary myelofibrosis.^1^ MPNs are characterized by clonal expansion of abnormal hematopoietic stem cells.^1^ In about 50–60% of patients with ET, *JAK2* V617F mutation is identified, followed by *CALR* and *MPL* mutations^2^ The *JAK2* V617F mutation causes constitutive activation of the JAK2-STAT tyrosine kinase signal transducers that mediate intracellular signals from different cytokine receptors and affect gene transcription, cell cycle regulation, cell differentiation and apoptosis.^3^ In about 20% patients no mutation is identified which does not preclude ET diagnosis, the so-called triple-negative patients.^4^

*JAK2* V617F mutation is associated with clonal hemopoiesis in MPN leading to development of the hematologic disease. However, cell clones with *JAK2* V617F are associated with multiple cardiovascular diseases: atherosclerosis and aortic thrombosis leading to ischemic stroke, coronary artery disease and heart failure, pulmonary hypertension, venous thrombosis, and aortic aneurysm.^5^ Furthermore, clonal hematopoiesis of indeterminate potential, defined as the presence of an expanded somatic blood-cell clone in persons without any hematologic abnormalities, is common among older persons and is associated with nearly a doubling in the risk of coronary heart disease in humans and with accelerated atherosclerosis in mice.^6^

We have previously reported that the increase in carotid artery stiffness and pulse wave velocity over the four-year observation period was much more pronounced in high-risk patients with *JAK2* V617F ET than in the control group.^7^ In the same cohort, we further determined the burden of the *JAK2* V617F mutation at the beginning and at the end of the four-year observation period and correlated changes in the mutation burden with the coronary artery calcium score. Our hypothesis was that the *JAK2* V617F mutation burden would be correlated with the coronary calcium score.

## Patients and methods

### Study design

The study design was described previously.^7,8^ Briefly, among 61 patients with *JAK2 V617F* positive ET who did not have clinically evident atherosclerotic disease, 40 participated at the first visit and of these 36 at the second visit after four-year time period. The control group consisted of 42 healthy control subjects participated at the first visit and 38 at the second visit. The study was approved by the Committee of Medical Ethics of the Republic of Slovenia (No. 154/05/12 and No. 0120-428/2017/4). The study has been registered at ClinicalTrials.gov PRS: Protocol Section NCT03828422.

### Baseline characteristics

At the first visit and at the fourth-year follow-up visit we physically examined the participants, measured their height, weight, waist circumference and blood pressure. The participants completed a structured questionnaire about personal and family medical history, medication and risk factors for cardiovascular disease.

Blood was drawn at the first and at the fourth-year follow-up visit for blood cell count, electrolytes, urea, creatinine, liver function tests and lipid profile. Inflammatory markers, i.e., high sensitivity C-reactive protein, interleukins IL-1, IL-6, IL-8, IL-10, tumor necrosis factor -alpha (TNF-α), P-selectin, vascular adhesion molecule -1 (VCAM-1) and von Willebrand factor (VWF-A2) were measured at the second visit.

### *JAK2* V617F mutation burden

*JAK2* V617F allele burden was determined in DNA extracted from granulocytes in peripheral blood, from samples collected at the Hematology Department, UMC Ljubljana at the time of the first visit, and from samples and the four-year follow-up visit. Real-time quantitative polymerase chain reaction (qPCR) was done with double-dye oligonucleotide hydrolysis, using Ipsogen JAK2 MutaQuant Kit, Qiagen (ZDA).^9^ Allele burden was calculated from the standard curves and was defined as the percentage of *JAK2* V617F mutated alleles in total *JAK2*.

At the first visit we analyzed 28 blood samples, as eight patients did not have their blood samples collected for allele burden determination. Blood samples were collected from all patients at the second visit. In total, we had 28 pairs of samples taken four years apart.

### Coronary calcium

The Biograph M 128-row PET-CT scanner (Siemens, Erlangen, Germany) was used for coronary artery calcium scanning. Scanning was done in sustained breath hold, from the carina to the base of the heart. We used a non-contrast protocol with sequential, prospective ECG triggering. Rotation time was 0.33 sec, tube voltage 120 kV, CARE Dose 4D and slice thickness 3 mm, with no slice overlap. Post-processing was done on a Syngo Leonardo workstation. The coronary calcium burden was expressed as the Agatston score.^10^ Measurements were done tree times for each visit and the average value was used for analysis.

### Statistical analysis

Variables were presented as mean and standard deviation (SD) or as median and interquartile range (IQR) when asymmetrically distributed. Paired versions of statistical tests were used when comparing study group in time. Counts were used for discrete variables, and differences between groups were assessed by Fisher exact test. Spearman correlation coefficient was used to calculate monotonic correlation between different parameters. All *p*-values were two-sided and *p*-values of < 0.05 were considered statistically significant.

## Results

### Patients and baseline characteristics

We included 36 subjects (12 male and 24 female) with ET and 38 control subjects (14 male and 24 female). The patient baseline characteristics are shown in Table 1.

**TABLE 1.** Baseline characteristics of patients with *JAK2* V617F positive essential thromocythemia (ET) and of control group at the first visit and at the second visit (body mass index, BMI)

	**FIRST VISIT**			**SECOND VISIT**			**Patient group**	**Control group**

							**First *vs*. second visit**	**First *vs*. second visit**
	Patient group	Control group	p-value	Patient group	Control group	p-value	p-value	p-value
**Age (years)**	55.11 (13.40)	59.07 (12.02)	0.186	58.36 (13.44)	62.08 (11.99)	0.214	-	-
**Sex (M/F)**	12/24	14/24	0.811	12/24	14/24	0.754	-	-
**BMI (kg/m2)**	25.22 (3.65)	27.27 (4.64)	0.038	26.13 (4.66)	27.54 (4.60)	0.195	0.021	0.184
**Waist (cm)**								
Male	94.6 (11.1)	102.2 (10.4)	0.086	98.4 (11.2)	104.0 (12.7)	0.260	0.014	0.201
Female	89.5 (9.1)	89.3 (14.2)	0.965	93.1 (11.1)	93.6 (15.0)	0.899	0.086	0.005
**Systolic blood pressure (mmHg)**	140 (22)	134 (14)	0.219	144 (19)	141 (20)	0.615	0.134	0.018
**Diastolic blood pressure (mmHg)**	81 (9)	82 (11)	0.870	86 (12)	89 (10)	0.247	0.044	< 0.001
**Smoking**			0.267			0.403	0.892	0.924
Current	5/36	3/38		4/36	3/38			
Former	10/36	6/38		12/36	8/38			

BMI = body mass index; M/F = male/fimale

Laboratory tests at the second visit are shown in Table 2. No correlation of the laboratory parameters with *JAK2* V617F mutation burden was found.

**TABLE 2.** Laboratory parameters of patients with *JAK2* V617F positive essential thromocythemia (ET) and of the control group at the second visit after 4-year follow-up. Means and standard deviations are given for the normally distributed data, medians and interquartile range are given for non-normally distributed data

**THE SECOND VISIT**	**ET PATIENTS (n = 36)**	**CONTROL GROUP (n = 38)**	**COMPARISON BETWEEN GROUPS (p-value)**
^2^Glucose (mmol/l)	5.00 (4.60–5.40)	5.00 (4.70–5.60)	0.565
^2^Creatinine (μmol/l)	76.10 (63.85–85.85)	71.30 (62.45–83.55)	0.351
^1^Total cholesterol (mmol/l)	5.00 (1.05)	5.33 (0.93)	0.163
^1^HDL-cholesterol (mmol/l)	1.45 (0.59)	1.66 (0.53)	0.118
^1^LDL-cholesterol (mmol/l)	2.66 (0.89)	2.94 (0.82)	0.168
^1^Triglycerides (mmol/l)	1.97 (0.89)	1.61 (0.80)	0.850
^1^Leukocytes (10^9^/L)	7.86 (2.83)	6.54 (1.63)	0.016
^1^Red blood cells (10^12^/L)	4.42 (0.69)	4.84 (0.42)	0.002
^1^Haemoglobinb	133 (15)	145 (12)	0.001
^1^Platelets (10^9^/L)	524.56 (218.67)	250.38 (60.05)	< 0.001
^1^Lymphocytes (10^9^/L)	1.88 (0.90)	2.02 (0.72)	0.195
^1^Mixed cells (10^9^/L)	0.71 (0.29)	0.59 (0.20)	0.067
^1^Neutrophils (10^9^/L)	4.81 (1.97)	3.93 (1.31)	0.031
^1^IL-1 (ng/L)	43.26 (4.98)	34.59 (6.48)	< 0.001
^1^IL-8 (ng/L)	28.89 (8.45)	20.64 (9.51)	< 0.001
^1^P-selectin (ug/L)	76.24 (19.54)	43.33 (13.36)	< 0.001
^1^VCAM-1 (mg/L)	1.17 (0.52)	0.72 (0.26)	< 0.001
^2^IL-6 (ng/L)	8.70 (7.70–9.38)	6.80 (6.28–7.40)	< 0.001
^2^IL-10 (ng/L)	5.65 (0.33–9.35)	5.25 (0.00–7.90)	0.417
^1^TNFa (ng/L)	43.90 (7.34)	37.60 (9.15)	0.002
^2^VWF-A2 (ng/L)	231.50 (199.25–256.25)	195.00 (167.50–213.00)	< 0.001
^2^hs-CRP (mg/L)	0.87 (0.50–2.16)	0.91 (0.55–4.43)	0.314

Comparisons between groups were tested by Student's t-test or the Mann-Whitney test^2^

HDL = high-density lipoprotein; IL = interkeucin; ldl = low-density lipoprotein; TNF-α = tumor necrosis factor –alpha; VCAM-1 = vascular adhesion molecule -1; VWF-A2 = von Willebrand factor -A2

### *JAK2* V617F allele burden

The average *JAK2* V617F allele burden at the first visit (n = 28) was 28.57% (SD 20.45%) and at the four-year follow-up visit (n = 36) 15.92% (SD 15.42%); p = 0.001. Over the four-year observation period *JAK2* V617F allele burden decreased in 19 patients, did not change in five patients and increased in four patients. Overall, the allele burden decreased significantly.

In the subgroup of patients where the allele burden decreased, the average *JAK2* V617F allele burden at the first visit was 37.93% (SD 15.57%) and at the fourth-year follow-up visit 19.65% (SD 14.86%), p < 0.001. In the subgroup of patients where allele burden increased or stayed the same, the median *JAK2* V617F allele burden at the first visit was 0.00% (0.00 – 15.93) and at the four-year follow-up visit 0.00% (0.0 – 31.55), p = 0.068.

In the control group *JAK2* V617F allele burden was measured only at the four-year follow-up visit (median 0.00% (IQR 0.00–0.00)).

### Coronary calcium

Table 3 presents coronary calcium burden of patents and control subjects at the first and at the four-year follow-up visit. The ET and control group did not differ in the Agatston score at both visits (p = 0.252 at the first visit and p = 0.954 at the four-year follow-up visit). The coronary calcium Agatston score increased slightly, but significantly in both groups: in the ET group from 0 (IQR 0–8.6) to 0.6 (IQR 0–40.3), *p* = 0.009 and in the control group from 0 (0–8.6) to 2.6 (0–30.1), p < 0.001. Overall, there was no correlation between the *JAK2* V617F allele burden and the calcium burden of coronary arteries (at the first visit r_s_ = 0.182, *p* = 0.355 and at the fourth-year follow-up visit r_s_ = 0.161, *p* = 0.355).

**TABLE 3.** Coronary calcium burden expressed as Agatston score of patients with *JAK2* V617F positive essential thromocythemia (ET) and control subjects at the first visit and at the fourth-year follow-up visit and the correlation with *JAK2* V617F allele burden for the ET patient group

	**FIRST VISIT**			**SECOND VISIT**			**Patient group First *vs*. second visit**	**Control group First *vs*. second visit**	**Correlation with *JAK2* V617F allele burden, p-value**	

	**Patient group**	**Control group**	**p-value**	**Patient group**	**Control group**	**p-value**	**p-value**	**p-value**	**First visit**	**Second visit**
**Agatston score**	0 (0–8.6)	0 (0–8.6)	0.525	0.6 (0–40.3)	2.6 (0–30.1)	0.954	0.009	< 0.001	0.191	0.069
**Calcium burden LM**	0 (0–0)	0 (0–0)	0.359	0 (0–1.2)	0 (0–0)	0.274	0.014	0.953	0.700	0.657
**Calcium burden LAD**	0 (0–2.7)	0 (0–2.68)	0.581	0 (0–32.5)	0 (0–21.3)	0.861	0.435	< 0.001	0.243	0.069
**Calcium burden LCX**	0 (0–0)	0 (0–0)	0.630	0 (0–0)	0 (0–0)	0.825	0.208	0.204	0.433	0.074
**Calcium burden RCA**	0 (0–0)	0 (0–0)	0.245	0 (0–0.8)	0 (0–0)	0.676	0.074	0.012	0.700	0.045

LAD = left anterior descending artery; LCX = left circumflex artery; LM = the left main coronary artery; RCA = right coronary artery

**FIGURE 1.**
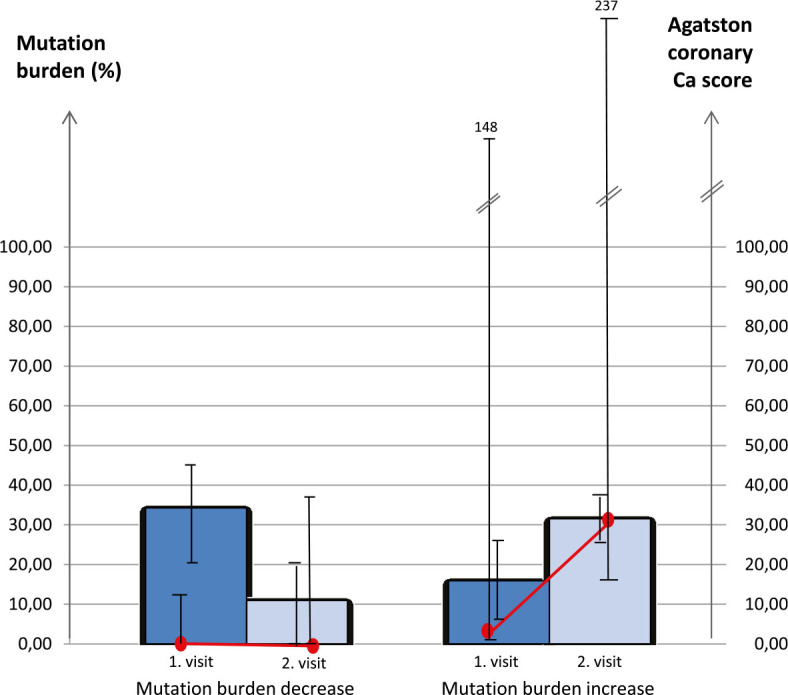
Change in *JAK2* V617F mutation burden and Agatston coronary calcium score over the 4-year observation period in patients whose *JAK2* V617F mutation burden increased (left-side columns) and in patients whose mutation burden decreased (right-side columns). Bar charts represent the mean mutation burden (%), and line graphs represent the median Agatston coronary calcium score.

In the subgroup of patients with ET, in which the *JAK2 V617F* mutation burden decreased, the coronary calcium score remained low without a change. However, in the patients in whom the *JAK2 V617F* mutation burden increased, the coronary calcium score also increased (Figure 1).

No differences were found in inflammatory parameters between the subgroup with increased and the one with decreased *JAK2 V617F* mutation burden. All measured inflammatory parameters (IL-1, IL-8, P-selectin, VCAM-1, IL-6, TNFα and VWF-A2) were elevated in both subgroups of patients with ET. On the other hand, IL-10 which is an anti-inflammatory parameter, was in normal range in both subgroups.

## Discussion

*JAK2* V617F mutation is the predominant mutation in MPNs and also the mutation most strongly associated with cardiovascular disease risk.^11^ It causes constitutive activation of JAK/STAT signaling which promotes expression of inflammatory cytokines, reactive oxygen species, production of oxidative low-density lipoproteins and formation of foam cells. This creates a chronic inflammatory state as a driving force for atherosclerosis.^12,13^

It is known that patients with *JAK2* V617F positive MPNs have accelerated atherosclerosis, higher incidence of acute coronary syndrome and other cardiovascular events.^14^ Experimental studies on animal models elucidated the pathophysiologic mechanisms that underlie increased cardiovascular disease risk in MPNs. Wang *et al.*, experimenting on mice, showed that *JAK2* V617F mutation promotes neutrophil infiltration and leukocytes attachment to the vascular wall, impairs macrophage function, accelerates atherosclerotic lesion formation with larger plaques which have unstable necrotic cores.^15,16^ In rabbits, Yang *et al.* found that JAK2 inhibitor blocks upregulation of inflammatory mediators, decreases plasma triglycerides, total cholesterol, and LDL, enhances HDL-C and reduces formation of atheromatous plaque.

Association between *JAK2* V617F mutation and atherosclerosis is being more thoroughly investigated in the last decade, however, little is known about correlation between allele burden and CVS risk. Our study examined the *JAK2* V617F allele burden in correlation with coronary artery calcium burden and inflammatory mediators in patients with high-risk ET. We found no overall correlation between the *JAK2* V617F mutation burden and the coronary artery calcium score. On the contrary, the recent publication by Nordheim Solli *et al.* showed that there is a significant association between the variant allele fraction in the upper quartile (≥ 52%) and severe coronary calcifications in patients with MPNs. The study had limited statistical power to focus on MPN subgroups, though, and it did not specifically address patients with ET.^17^ However, in our study with high-risk ET patients coronary calcium burden increased during the four-year follow-up in the small subgroup of patients whose mutation burden increased as well. This, though not statistically significant, was to our knowledge observed for the first time in the developing field of research in MPN, atherosclerosis and coronary calcium burden. Conversely, in the subgroup of our patients where mutation burden decreased during the observation time, there was no progression in coronary artery calcium burden.

In the subgroup of patients where mutation burden increased, the average *JAK2* allele burden at the first visit was lower in comparison to the subgroup where allele burden decreased However, they reached comparable levels at the second visit (Figure 1). Six out of 19 patients (32%) whose allele burden decreased and three out of nine patients (33%) whose allele burden increased or stayed the same had undergone a change in hematologic therapy. Changes in therapy were all different and overall did not seem to have any influence on allele burden. An uneven change in kinetics of the *JAK2* V617F allele burden over time was observed also by Antonioli *et al.*^19^

The mutation burden was independent of patients' age which was previously observed by Kittur *et.al.*^20^ Also, consistent with previous data^19,20,21^, we found no correlation of *JAK2* V617F mutation burden with levels of erythrocytes, leukocytes or platelets. Previous research found an association of *JAK2* V617F allele burden with increased CRP levels in patients with ET.^22^ In contrast, we did not find any correlation between allele burden, CRP and other inflammatory mediators. Yet, there was a negative correlation between *JAK2* V617F allele burden and IL-10 (r = −0.333, p = 0.047), which is an anti-inflammatory factor.

Among chronic myeloproliferative disorders, ET is characterized by the greatest heterogeneity in clinical profile, as well as in cellular and molecular levels.^19^ Autonomous activation of the JAK-STAT pathway in ET patients is progressively increased with the amount of mutant allele.^19^ Splenomegaly was significantly more frequent when the mutation burden was over 50%, and symptoms due to microvascular disease were present when the mutant allele level was over 25%. Also, a 3-fold greater risk of arterial thrombosis was found in those patients.^19^ In our study group, the overall *JAK2* V617F mutation burden was relatively low. Therefore, a lack of correlation between overall allele burden and coronary artery calcium burden or inflammatory mediators might be accounted for by confounding factors overshadowing the relatively low burden of mutant alleles.

*JAK2* V617F associated abnormalities are more common in patients with polycythemia vera or primary myelofibrosis where allele burden is much higher than in ET.^23,24^ Correlations between *JAK2* V617F allele burden and clinical features in ET are not as definite. Available data about the clinical and prognostic importance of the *JAK2* V617F mutation in patients with ET are still incomplete and sometimes even controversial.^21,25^

In some reports, higher mutated *JAK2* allele burden was associated with increased blood counts and hemoglobin^26,27^ but this was not confirmed by others.^28,29,30^ Thrombotic risk was elevated in patients with ET.^26,31^ Also, higher mutant allele burden together with histology classification was associated with disease progression to primary myelofibrosis.^32^

All ET patients in our study were identified as high risk for thrombotic complications and were treated accordingly. Acetylsalicylic acid (ASA) was started in all except if they had an indication for anticoagulation therapy. Low-dose ASA significantly reduces thrombotic complications in ET patients^33^ and may have some anti-inflammatory effect in the setting of atherosclerosis.^34^ Anagrelide was the most common choice of drug for platelet reduction, followed by hydroxyurea. Anagrelide successfully achieves hematologic response in ET^35,36^, however, it does not have any impact on *JAK2* allele burden.^37^ Hydroxyurea is a preferable choice to anagrelide in older patient population with similar effectiveness as anagrelide but with less cardiovascular side effects^33,36^, also not affecting the *JAK2* allele burden.^38,39^ Interferon is a second line treatment choice, that can prolong the time to disease progression, may prolong survival in MPNs and ET and often significantly reduces the *JAK2* allele burden.^40,41,42^ However, we used interferon for a short period in only two patients, in whom it did not lead to a significant change in *JAK2* burden. Ruxolitinib, a *JAK1/2* inhibitor, though not a standard of care in ET, was used in two patients. Ruxolitinib was shown to affect *JAK2* burden in patients with ET and could lead to molecular remissions.^43,44^ However, again as in patients on interferon, we could not draw any conclusions due to the low patient numbers. Thus, the treatment landscape of patients in our study was very heterogenous and primarily focused on hematologic responses with most probably no impact on *JAK2* allele burden.

### Study limitations

The main limitation of our study is the small number of participants. As we decided to determine *JAK2* V617F allele burden after our initial study, blood samples from eight participants were not collected at the first visit and we were unable to determine their initial *JAK2* V617F allele burden.

A minor limitation is that all participants were not examined at the exact time interval between both visits, however, the time difference varied at most for a few weeks.

In conclusion, our study, contrary to expectation, showed a decrease of the average *JAK2* V617F allele burden in patients with high-risk ET during four-year observation period. However, in the small subgroup of four patients whose *JAK2* V617F mutation burden increased the Agatston coronary calcium score increased as well but the significance of this finding cannot be calculated due to the small sample. This preliminary finding, which should be interpreted with caution and validated in a larger study, is in line with the emerging evidence that the *JAK2* V617F mutation is a non-classical risk factor for cardiovascular disease.
